# Supplementary material for: Aromatic interactions with membrane modulate human BK channel activation
Source: eLife. 2020 Jun 29;9:e55571. doi: 10.7554/eLife.55571 (PMC7371421; doi:10.7554/eLife.55571)
Supplement: Supplementary file 1. — The position of insertion (S337) is colored red. [file elife-55571-supp1.docx]

**Supplementary Table 1.** Effects of C-linker insertion/deletion mutations on the voltage required for half channel activation (V_0.5_) of hSlo1 in absence of Ca^2+^ and Mg^2+^ (data extracted from: Niu et al, 2004). The position of insertion (S337) is colored red.

| **Mutation** | **V_0.5_ (mV)** | **C-linker Sequence** |
| --- | --- | --- |
| -3 | 121 | RKKYGG - - - AVSGRK |
| -1 | 132 | RKKYGGSY- AVSGRK |
| WT | 184 | RKKYGGSY**S**AVSGRK |
| +3 | 210 | RKKYGGSY**S**AAGAVSGRK |
| +6 | 246 | RKKYGGSY**S**AAGAAGAVSGRK |
| +12 | 326 | RKKYGGSY**S**AAGAAGAAGAAGAVSGRK |
